# EPIDEMIOLOGIC GEROSCIENCE INNOVATIONS AT THE NEXUS OF REPRODUCTIVE AGING AND WOMEN’S HEALTH

**DOI:** 10.1093/geroni/igad104.1437

**Published:** 2023-12-21

**Authors:** Michelle Shardell

## Abstract

Signs of reproductive aging in women usually begin in women’s 30s and co-occur with declining circulating estrogen concentrations and irregular menstrual cycle onset. Menopause, defined by cessation in menstruation for ≥1 year, occurs on average at age 51 years, but about 1% experience menopause before age 40. Menopause and reproductive aging contribute to multiple health conditions such as certain cancers; however, a less studied condition is the genitourinary syndrome of menopause (GSM). GSM signs and symptoms include vulvovaginal atrophy, vaginal dryness, urinary tract infections, and more. Additionally, menopause leads to increased risk of osteoporosis, dementia, and other aging-related conditions. Given the multifactorial implications of reproductive aging in women’s health, an interdisciplinary geroscience-guided framework for epidemiology of aging is needed to identify biological contributors and consequences of reproductive aging to develop healthspan-enhancing interventions. In this symposium, we present five new developments across multiple stages of reproductive aging, consistent with meeting theme “Building Bridges. Catalyzing Research. Empowering All Ages”. Among women aged 35-60 years, we assess the role of cervical “inflamm-aging” in GSM; diabetes and vaginal dysbiosis associations with GSM; and vaginal microbial and metabolic signatures of urinary conditions/dysfunction. Among women aged ≥65 years, we examine associations of post-hip fracture sex hormones with physical recovery and links between age and type of menopause with Alzheimer’s disease. Building on the 2021 Journals of Gerontology Series A special issue on intersections of reproductive and aging biology, we discuss opportunities to advance reproductive aging and women’s health research by combining molecular assessment with rigorous epidemiology. This is a collaborative symposium between the Epidemiology of Aging, Geroscience, and Women’s Issues Interest Groups.

